# A Modelling Framework to Assess the Effect of Pressures on River Abiotic Habitat Conditions and Biota

**DOI:** 10.1371/journal.pone.0130228

**Published:** 2015-06-26

**Authors:** Jochem Kail, Björn Guse, Johannes Radinger, Maria Schröder, Jens Kiesel, Maarten Kleinhans, Filip Schuurman, Nicola Fohrer, Daniel Hering, Christian Wolter

**Affiliations:** 1 Department of Biology and Ecology of Fishes, Leibniz-Institute for Freshwater Ecology and Inland Fisheries, Berlin, Germany; 2 Department of Hydrology and Water Resources Management, Kiel University, Kiel, Germany; 3 Department of Aquatic Ecology, University of Duisburg-Essen, Essen, Germany; 4 Department of Physical Geography, Utrecht University, Utrecht, The Netherlands; Virginia Commonwealth Univ, UNITED STATES

## Abstract

River biota are affected by global reach-scale pressures, but most approaches for predicting biota of rivers focus on river reach or segment scale processes and habitats. Moreover, these approaches do not consider long-term morphological changes that affect habitat conditions. In this study, a modelling framework was further developed and tested to assess the effect of pressures at different spatial scales on reach-scale habitat conditions and biota. Ecohydrological and 1D hydrodynamic models were used to predict discharge and water quality at the catchment scale and the resulting water level at the downstream end of a study reach. Long-term reach morphology was modelled using empirical regime equations, meander migration and 2D morphodynamic models. The respective flow and substrate conditions in the study reach were predicted using a 2D hydrodynamic model, and the suitability of these habitats was assessed with novel habitat models. In addition, dispersal models for fish and macroinvertebrates were developed to assess the re-colonization potential and to finally compare habitat suitability and the availability / ability of species to colonize these habitats. Applicability was tested and model performance was assessed by comparing observed and predicted conditions in the lowland Treene River in northern Germany. Technically, it was possible to link the different models, but future applications would benefit from the development of open source software for all modelling steps to enable fully automated model runs. Future research needs concern the physical modelling of long-term morphodynamics, feedback of biota (e.g., macrophytes) on abiotic habitat conditions, species interactions, and empirical data on the hydraulic habitat suitability and dispersal abilities of macroinvertebrates. The modelling framework is flexible and allows for including additional models and investigating different research and management questions, e.g., in climate impact research as well as river restoration and management.

## Introduction

River biota are affected by anthropogenic pressures acting at different spatial scales, ranging from global climate and catchment scale hydrological changes [1], catchment scale water quality pressures including point and diffuse source pollution such as nutrients, pesticides, and fine sediment [2] to reach-scale hydromorphological alterations such as channelization. In addition to these changes in abiotic habitat conditions, the biota of rivers depends on the species pool available for re-colonization. Sensitive species of the natural species pool are often rare or extinct in the catchment or region, limiting the number of source populations. Moreover, migration barriers restrict (re-)colonization of newly restored habitats or river reaches depopulated by natural or human-caused calamities [3].

Several empirical studies indicate that large-scale pressures, as measured by proxies such as catchment land use, can be more important in shaping macroinvertebrate and fish communities compared to pressures at smaller spatial scales [4–9]. Large-scale pressures such as nutrient or fine sediment loads and water pollution can even limit macroinvertebrate assemblages [10–12] and, hence, potentially govern river biota and constrain the effect of reach-scale river restoration. It is inherently difficult to infer causal relationships based on empirical analysis because variables often show high collinearity and are proxies for different pressures. Complementary modelling could help to assess the importance of different pressures by coupling models that predict the effect of these pressures on abiotic habitat conditions and biota.

Most modelling approaches for predicting the biota of rivers focus on the hydromorphological conditions at the reach or river segment scale and on habitat suitability. In the widely used microscale habitat models, the hydraulic microhabitat conditions (e.g., flow velocity and depth and substrate) are predicted using a hydraulic model, and habitat suitability is assessed based on environmental-biological relationships [13,14]. In mesoscale habitat models, channel features such as riffles are mapped, probability density functions of microhabitat conditions for each mesohabitat are derived from statistical analysis, and meso- as well as microhabitat descriptors are used to assess habitat suitability based on environmental-biological relationships [15]. These habitat models are mainly based on empirical relationships, and more mechanistic models have been developed only recently [16]. However, these approaches consider static channel geometries and neglect dynamic long-term morphological changes that affect the habitat conditions. Furthermore, there are only a few modelling frameworks that also consider large-scale pressures at the global, catchment or river network scale in addition to the reach and segment scale habitat conditions. Kiesel et al. [17] linked hydrological and 1D hydrodynamic models to predict discharge at the catchment scale and the resulting water level at the downstream end of a study reach. The respective flow and substrate conditions in the study reach were predicted using a 2D hydrodynamic model, and the suitability of these habitats for macroinvertebrates was assessed. Based on this approach, Jähnig et al. [18] also coupled a hydrological and 1D hydraulic model but used it to predict 1D flow conditions and sediment load at the river segment scale, which in turn were used to develop a species distribution model to exemplarily predict the presence of one macroinvertebrate species. These applications of integrated modelling approaches focus on macroinvertebrates, while studies comparing different organism groups (e.g., fish and macroinvertebrates) are lacking. Furthermore, we are not aware of any modelling framework considering not only habitat availability and suitability but also the re-colonization potential, which is affected by limited source populations due to depleted species pools and migration barriers. However, the effect of regional species pools and dispersal limitations on species composition has been identified as one of the fundamental questions in ecology [19] and modelling of river biota [16].

The overall aim of this study was to develop a modelling framework of coupled models to assess the effect of different pressures on abiotic habitat conditions and the biota of rivers, partly based on the work of Kiesel et al. [17]. The main changes to the modelling framework of Kiesel et al. [17] were to (i) include models to predict the effect of discharge changes on channel morphology, (ii) develop and implement dispersal models to assess the re-colonization potential in addition to modelling habitat suitability, and (iii) consider fish and macroinvertebrates to test the applicability of such a modelling framework for different organism groups. The main objectives of the present methodological paper were to present the extended modelling framework, illustrate its application using a case study, and discuss its potential and limitations.

## Modelling Framework

**Fig 1:**
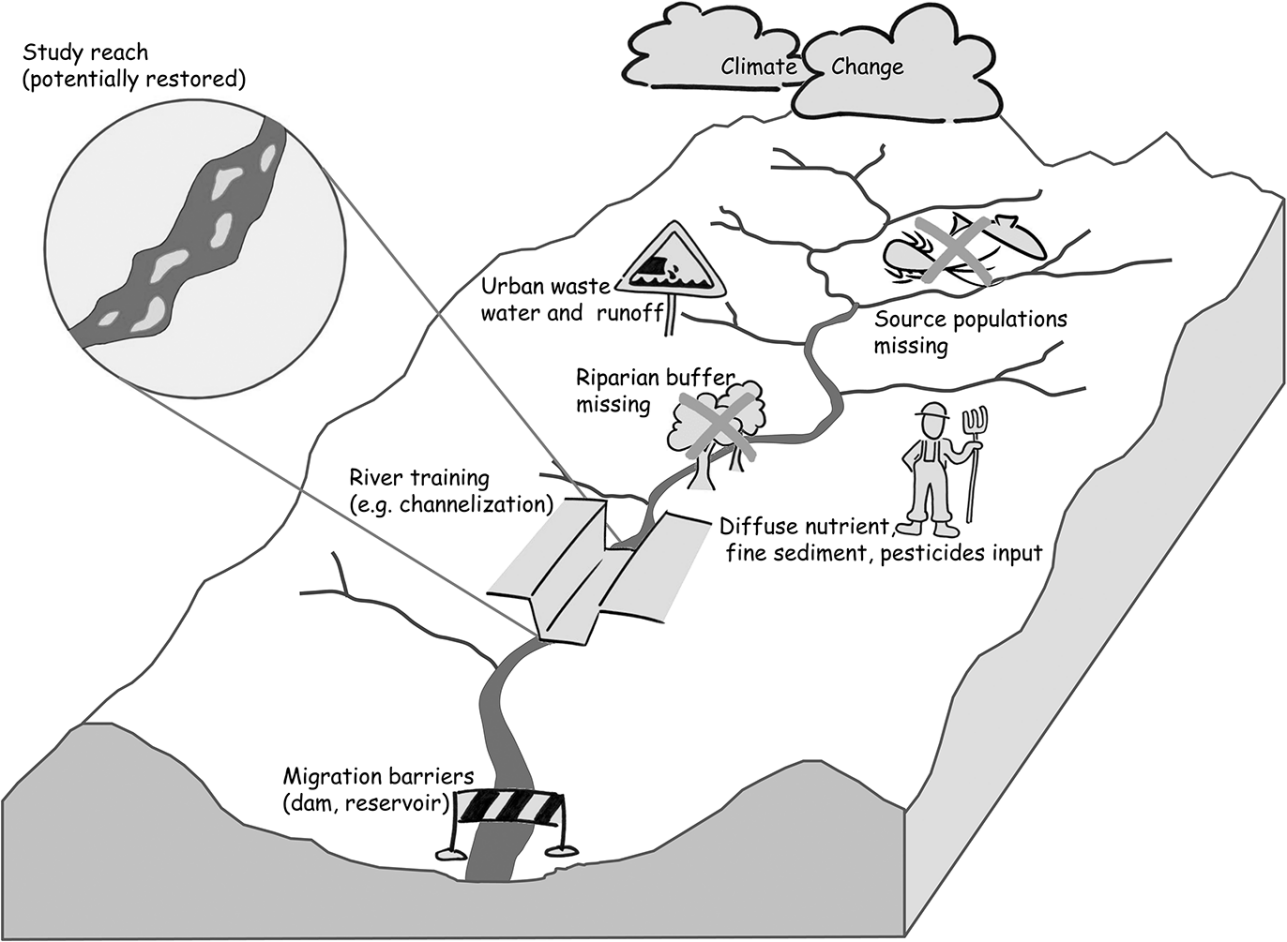
Local abiotic habitat conditions and river biota are affected by pressures at different spatial scales that potentially constrain reach-scale biota and restoration.

**Fig 2:**
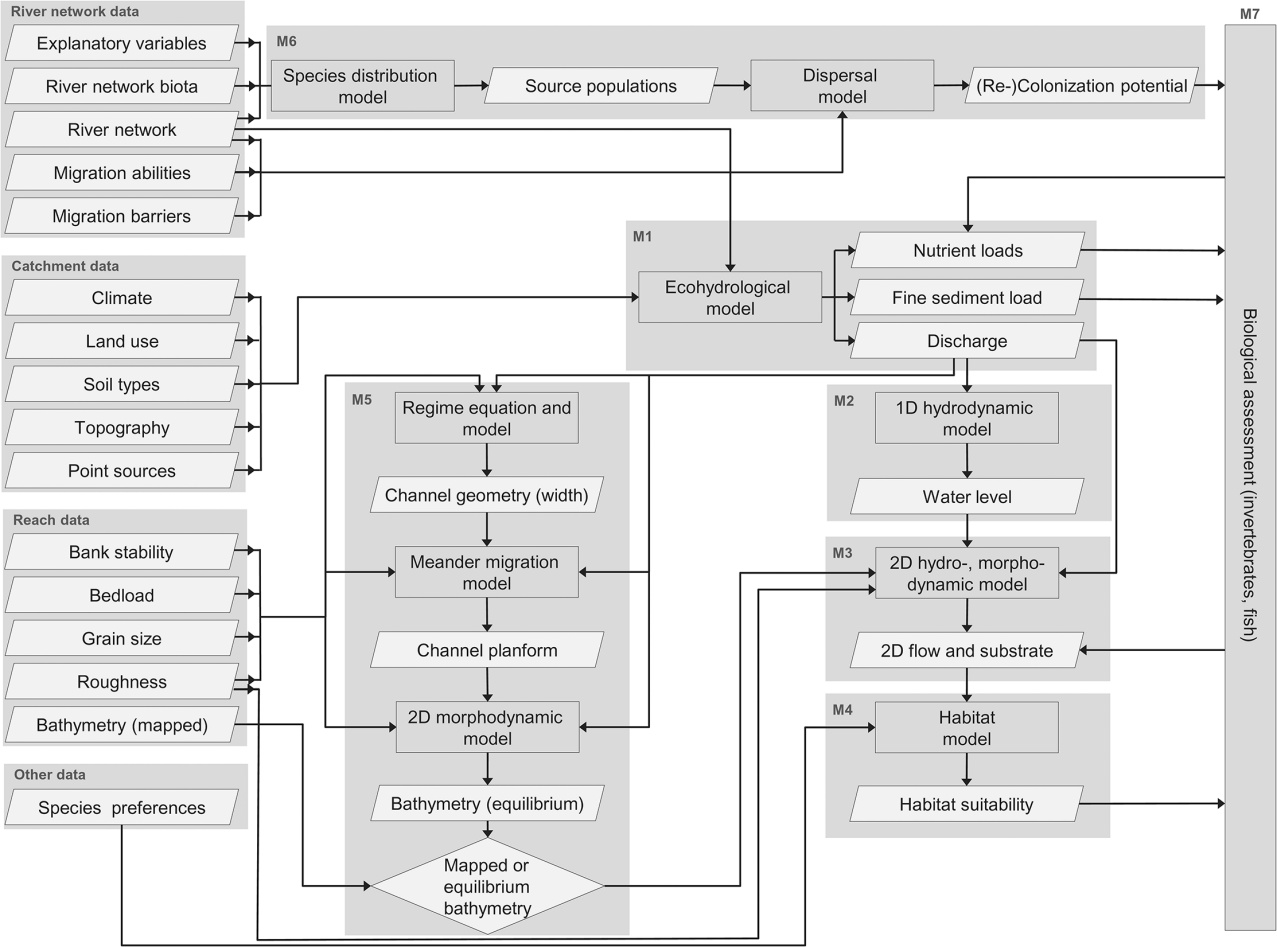
Flow chart of the modelling framework. Models are coupled to predict how the effect of catchment-scale pressures propagate to the river network and reach scale and to assess the effect of different pressures on abiotic habitat conditions and biota of rivers. Different symbols are used for models (rectangles), input and output data (parallelograms), and decisions (diamond symbol).

In the modelling framework, the following pressures are considered: (i) discharge changes and (ii) water quality aspects (nutrient and fine sediment loads) due to climate and land-use changes at the global and catchment scale, respectively, (iii) habitat fragmentation and migration barriers at the river network scale, and (iv) reach-scale hydromorphological changes and habitat alterations (Fig 1). The pressures affect the abiotic habitat conditions and biota at the river network scale, and species become locally extinct, limiting the number of source populations and–in addition to migration barriers–the re-colonization potential at the reach scale (Fig 1). Different hydrological, hydraulic, morphological, and biological models are coupled (Fig 2) to assess the effect of different pressures on the abiotic habitat conditions and biota of a study reach given the (limited) re-colonization potential (Fig 1).

First, an ecohydrological model is used to predict water quantity and quality (Fig 2M1). Catchment data on climatic variables, land use, soil types, topography, and point sources are used to predict discharge, suspended sediment, and nutrient loads along the river network. Second, a 1D hydrodynamic model is applied to develop a rating curve, which gives the water level at the downstream end of the study reach (Fig 1) for a given discharge (Fig 2M2). Third, a 2D hydro- and morphodynamic model predicts the hydraulic habitat conditions (e.g., flow velocity, depth, and shear stress) and grain size distribution of non-cohesive mineral substrates in the study reach based on the mapped channel bathymetry for different discharges and corresponding water levels at the downstream end (Fig 2M3). Fourth, the suitability of the modelled hydraulic habitat and substrate conditions for biota are assessed using habitat suitability models (Fig 2M4). These four models have already been exemplarily coupled for macroinvertebrates by Kiesel et al. [17].

The extended modelling framework presented here includes two additional types of models. Morphological models (Fig 2M5) were added for two reasons: First, the present channel morphology might be in disequilibrium and not adapted to the governing controls such as the imposed water discharge, sediment load, and sediment properties and, hence, not representative of the long-term morphological and habitat conditions. Second, alterations of these controls can cause channel instability (e.g., increased urban runoff or high flows due to climate and land-use change) [20]. These morphological changes in turn affect channel hydraulics, habitat conditions and suitability. Presently, there is no physical-based morphodynamic model available to predict the long-term evolution and dynamic equilibrium state (however, see recent progress towards such a model in Asahi et al. [21]). Alternatively, different aspects of channel morphology are modelled in three consecutive steps (channel geometry, planform, and bathymetry). Channel geometry (width, depth, and slope) can be predicted using (i) empirical regime equations that are derived from observed channel geometry [22–24]), (ii) regime models, consisting of an indeterminate set of equations that are numerically solved using an extremal hypothesis [25–29]), (iii) other, more physically based approaches that remain empirically calibrated [30,31] and, hence, are difficult to apply in the modelling framework. Channel planform changes of freely meandering rivers (meander migration and lateral channel dynamics) can be modelled using a meander migration model, in which bank retreat is caused by fluvial entrainment of bank material due to excess flow velocity [32–35] and mass failure due to excess water depth [36–38]. Virtually all models assume a constant channel width (inner bend bank accretion equals outer bend bank retreat) [39] and require channel width predicted by the regime equation or model (Fig 2M5) as a necessary input. Channel bathymetry can be modelled using standard 2D or 3D morphodynamic models.

Dispersal models for fish and macroinvertebrates were developed and implemented (Fig 2M6) to consider the species pool available for re-colonization of the study reach in addition to abiotic habitat conditions. Dispersal modelling consists of two steps: (i) identifying potential source populations in the river network using field sampling and/or species distribution models and (ii) modelling the dispersal of a species from potential source populations depending on its specific migration abilities and the presence of migration barriers. Finally, information on habitat suitability, water quality and physico-chemical aspects (e.g., nutrient loads), and re-colonization potential are combined to assess the effect of the different pressures on the abiotic habitat conditions and biota of a study reach (Fig 2M7).

It is neither feasible nor ecologically meaningful to model morphological changes and habitat suitability with the temporal resolution given by the ecohydrological model (usually daily time series). Based on the modelled hydrograph, morphologically and ecologically meaningful hydrological variables must be calculated to describe the flow regime. The morphological models need formative discharge as a necessary input, which is usually approximated by bankfull flow (see review in Soar and Thorne [40]). Because bankfull discharge by definition depends on channel cross-section form and cannot be derived directly from the modelled hydrograph, a flow regime variable has to be selected as a proxy, which is related best to bankfull discharge. In the ecological literature, magnitude, frequency, timing, duration, and flashiness are usually listed as ecologically meaningful flow regime variables. Moreover, high/low flow events are considered important in addition to mean values because they often serve as ecological bottlenecks [41–45]. The magnitude and frequency of extreme events can be considered by selecting discharges with a high and low probability of occurrence, respectively.

## Case-Study Application

### Case study design and area

The applicability of the modelling framework was tested in a lowland river in northern Germany (Treene) using the results of the baseline scenario of an environmental change study. In this paper, we tested if it is technically possible and feasible to couple the different models and assessed the performance of the models by comparing measured and modelled data for the present conditions. The results of environmental change scenarios (climate and land-use change) will be published in an upcoming paper.

The Treene River is a mid-sized (481 km^2^, non-tidal influenced) lowland river dominated by agricultural land use (80%). Naturally, it is heavily meandering and dominated by sandy substrate with some gravel patches [46]. The study reach is located in the epipotamal region [47], and hence, the fish assemblage is naturally dominated by stone loach (*Barbatula barbatula*), gudgeon (*Gobio gobio*), dace (*Leuciscus leuciscus*), and three-spined stickleback (*Gasterosteus aculeatus)* but also inhabited by fish species of the upstream hyporhithral region such as minnow (*Phoxinus phoxinus*) and downstream metapotamal reaches such as roach (*Rutilus rutilus*) and perch (*Perca fluviatilis*). Similarly, the natural macroinvertebrate community is rather diverse, dominated by burrowing species and species inhabiting lentic areas rich in organic material, but also includes rheophilic species and species preferring hard substrates such as gravel, large wood and macrophytes [46]. Presently, large parts of the river network are not affected by saprobic pollution (high or good state according to the PERLODES assessment method, http://www.fliessgewaesserbewertung.de/en/) but are in a moderate to poor hydromorphological state according to the standard assessment method (corresponding to the LAWA method described in Gellert et al. [48]). The river network is fragmented by 52 migration barriers (0.16 barriers km^-1^). One of the few near-natural reaches in the lower part of the catchment (54°35'2.65"N, 9°20'22.10"E, 227.0 km^2^), 260 m in length, was investigated as an analogue for the habitat conditions resulting from typical projects restoring natural channel dynamics at the reach scale. It was considered near-natural because it is heavily meandering, has a mean bankfull width (10.9 m) and depth (1.4 m) typical of a natural river with highly cohesive river banks (80–100% silt/clay content) and a bankfull discharge of 6.0 m^3^/s. It differs from its natural state because the bed material is pure sand with a D_50_ of 0.16 mm, while gravel patches are missing, and it is bordered by pasture instead of floodplain forest [46]. The mean discharge is 3.0 m^3^/s, and the average slope is approximately 0.045‰. The Treene was selected as the case-study area because hydromorphological and ecological research and modelling have long focused on gravel-bed rivers (e.g., Habersack et al. [49]), and we expected to learn more from an application in a sand-bed river. Access to the study site located on private property was kindly granted by the owner (please contact co-author Prof. Nicola Fohrer for details). The field studies did not involve endangered or protected species, and only invertebrate samples were taken (no vertebrates).

### Materials and methods

Semi-distributed **ecohydrological models** such as SWAT [50] predict discharge, water fluxes and water constituents (e.g., nutrients, sediment, pesticides) for parts of the (sub-) basins with equal properties. They are the most suitable for application in the modelling framework because they combine (i) reasonably fast computational times compared to high-resolution grid-based models with (ii) the capability to assess the impact of spatially explicit land-use changes, in contrast to lumped models, which aggregate input data within whole (sub-) basins. SWAT was set up and calibrated using climate data from four stations, discharge data from six hydrological stations in the Treene catchment, and daily measurements of nitrate concentrations from 10/2010 to 09/2012 at the catchment outlet. The model consisted of 108 subbasins, with one having its outlet at the gauging station downstream of the study reach for model calibration. Another subbasin had its outlet at the downstream end of the study reach to provide modelled discharge and nitrate loads for the hydrodynamic models (1D and 2D) and the macroinvertebrate habitat model, respectively. The model was run for the recent period 1997–2012, including a warm-up period of four years. Thus, the calibration period for discharge was set for 2001–2005 and the validation period for 2006–2012. The results of the regional climate model STAR [51] were used to quantify the baseline climate conditions assuming no increase in temperature during the modelling period (2021–2060). STAR is a statistical climate model for which 100 baseline simulations were available, resulting in 100 modelled hydrographs that were used to calculate (i) Q10 as a proxy for bankfull discharge used in the morphological models (i.e., the value of the flow duration curve exceeded 10% of the days) and (ii) mean monthly values for Q75, Q50, and Q25 as ecologically meaningful hydrological variables to describe the flow regime.

A large number of **1D hydrodynamic models** are available, and any of them could be used for application in the modelling framework [52–55]. We used HEC-RAS [55] to develop the rating curve for the downstream end of the study reach because an interface to SWAT has recently been developed [56]. For the HEC-RAS model, 42 cross-sections were mapped between the next gauging station, which was located approximately 3 km downstream, and the study reach. Roughness (Mannings n) was estimated based on measured flow velocity and depth and set to n = 0.03, which corresponds to the literature values [57]. The modelled river segment was subdivided at the confluence of the only main tributary, and discharge values were adapted for the upstream segment by subtracting the discharge of the tributary. The rating curve was based on modelled water surface elevations at the downstream end of the study reach for a range of discharges below bankfull assuming steady flow conditions in both segments.

**Channel geometry** was modelled using an empirical hydraulic geometry equation for lowland sand-bed rivers in northern Germany, which was based on field data for 14 near-natural reaches comparable to the study reach. Furthermore, field data on bankfull geometry and discharge data for nearby gauging stations were used to identify the flow regime variable best related to bankfull discharge, which was Q10. The MIANDRAS model [37] was selected to predict **channel planform** changes and dynamics because it considers both relevant migration processes (fluvial entrainment and mass failure). For model calibration, channel width was measured in the field, channel roughness was back-calculated based on measured flow velocity data, grain size was determined from dry sieving of bulk samples, and a time series of aerial photos (1953–2008) was used to derive empirical data on past channel migration rates. Bankfull was set to the discharge where wetted area in the 2D-hydrodynamic model sharply increased (6.0 m^3^/s), as recommended by Navratil et al. [58], which did correspond well with the proxy for bankfull discharge Q10 of 6.4 m^3^/s, which was predicted by the ecohydrological model. **Channel bathymetry** was modelled using Delft3D because it can predict local-scale spatial variations in flow conditions relevant for fish and macroinvertebrates without sensitivity to the grid resolution. The curvilinear grid was created from the mapped planimetric shape of the channel reach, and the mapped bathymetry (0.5 measurement points m^-2^) was used as the initial bed. Upstream discharge, downstream water level, and average bed material calibre were specified as in the two-dimensional model, and roughness was calibrated to obtain quasi-uniform flow for a given upstream discharge and downstream water level.

The FaSTMECH solver from the iRIC software was used as a **2D hydrodynamic model** because computational time is low despite (i) the large number of runs for monthly values of different flow regime variables and (ii) the high resolution of the computational grid of 0.25 m^2^, which was chosen to model hydraulics at a spatial scale relevant for macroinvertebrates. Moreover, it is freeware (http://i-ric.org/en/introduction). Morphodynamics and sediment sorting were not modelled for the Treene case study because sand was the only mobile substrate, and hence, any sediment transport and sorting would have had no effect on the modelling results of the habitat model for macroinvertebrates (HET).

Conventional **habitat models** quantify the suitability of a habitat for specific species based on habitat preference curves (mostly univariate relationships between species presence and single habitat variables such as water depth and velocity). However, developing habitat preference curves is data intensive, and several species do not show clear habitat preferences due to opportunistic resource use and broad environmental tolerances (e.g., roach and perch). Therefore, a fuzzy logic model [59,60] was used following Conallin et al. [14]. It considers uncertainty in assessing habitat preferences by not directly using measured continuous or categorical habitat variables (e.g., flow velocity) but fuzzy membership functions, which allow assigning a specific value to more than one class. More specifically, habitat suitability for fish was modelled using the GRASS GIS tool r.fuzzy.system [61], a free and open source implementation of Zadeh’s [62] and Mamdani and Assilian’s [63] fuzzy inference system within a geographical information system for large datasets that is entirely based on open source software. The fuzzy membership functions and logical rules were assigned by expert judgement based on the hydraulic habitat preferences (flow velocity and depth) of eight species occurring in the Treene catchment. Habitat suitability with respect to flow velocity and depth was calculated for each raster cell of the 2D hydrodynamic computational grid for monthly values of Q75, Q50, and Q25. Grid values were weighted by area and summed, resulting in the Weighted Usable Area (WUA, [64,65]), a single value for a whole study reach. However, it is not possible to deduce the presence or abundance of species from these habitat suitability models because information on the WUA that is necessary for a species to establish is missing, which also hampers the model validation using species presence data. Nevertheless, it was possible to qualitatively assess the effect of changes in habitat suitability (WUA) and water quality on species presence because they are in principle positively correlated.

For macroinvertebrates, the Habitat Evaluation Tool (HET) was used, which was developed for the modelling framework by Kiesel et al. [66]. HET uses empirical data on the abundance of macroinvertebrates on specific substrates and hence can indeed predict the presence and abundance of macroinvertebrates in a specific river reach based on the mapped or modelled substrate composition. In contrast to the seasonal changes of the habitat conditions for fish (e.g., monthly values of WUA), the presence and abundance of macroinvertebrate species cannot be modelled for time periods shorter than the reproductive cycle due to the limited temporal resolution of the empirical biological data used to develop the HET. The calibration dataset from the Treene study reach included a total of 80 substrate-specific samples (25 cm^2^, eight replicates taken from each out of ten different substrate types). Invertebrate abundance predicted by the habitat model (HET) was corrected according to dose-response relationships between water quality parameters and species abundance [66].

**Dispersal modelling** for fish is in its infancy, and the first spatially explicit dispersal model for fish *FIDIMO* has been developed by Radinger et al. [67] within the modelling framework. As with all dispersal models, it requires information on (i) the location of potential source populations (e.g., from species distribution models or sampling data), (ii) species-specific dispersal abilities [68], and, optionally, (iii) migration barriers. The species-specific probability of fish moving to up- and downstream river reaches is calculated starting from each spatially explicit source population. The output of FIDIMO is a map indicating the probability of occurrence in the raster cells of the river network, optionally including maps showing the upper and lower confidence limits, and hence considering the uncertainty in assessing the dispersal abilities based on empirical data of fish movement [68]. In contrast to fish, empirical data on the movement distances and migration abilities for macroinvertebrates are scarce, and information is mainly restricted to gross classifications of dispersal abilities [69]. Furthermore, the dispersal of merolimnic macroinvertebrates fundamentally differs from the unidirectional (up- and downstream) dispersal of aquatic fish and macroinvertebrate fauna due to the terrestrial life-stage. Therefore, least-cost modelling was considered the best suited because it allows accounting for differences in the permeability of the landscape and identifies the lowest accumulated (friction) cost between any raster cell and the source raster cells (source populations) [70,71].

For the dispersal modelling in the Treene river network, source populations were predicted using species distribution models (SDMs). The presence of eight fish species was modelled using Boosted Regression trees based on 81 fish samples and the following environmental data: hydromorphological conditions at the sampling sites and adjacent river reaches (hydromorphological survey data similar to the LAWA on-site survey described in Gellert et al. [48], including information on migration barriers) and topological data (Strahler and Shreve stream order and distance from the mouth). The probability of occurrence given by the SDMs was converted into binary presence/absence maps using a commonly used objective threshold given by the SDMs that maximizes the sum of sensitivity (true positive rate) and specificity (true negative rate) [72]. This threshold achieves the maximum correctly predicted presences and absences in the final binary output map. For 7 macroinvertebrate species, source populations were identified using the same approach as for fish based on 77 macroinvertebrate samples and environmental variables (hydromorphological survey data, land-use data).

The re-colonization potential for fish was quantified in FIDIMO [67] by calculating the species-specific share of the river network as well as the 95% confidence interval, which was predicted to be reachable (all grid cells considered reachable that were within 99% of the probability density function, i.e., 1% of the source populations were predicted to move further, upstream migration barriers considered, and the 3-year modelling period corresponded to the mean generation interval of the model species). The re-colonization potential for macroinvertebrates was quantified by calculating the species-specific share of the river network, which was predicted to be reachable after one life cycle (one year for most model species) using SDMs as source populations and the Cost Distance and Path Distance tools in ArcGIS [73,74]. After calculating the lowest accumulated cost for all raster cells, all cells below a specific cost threshold were classified as reachable. The model was calibrated for results to reflect movement distances reported in the literature or based on expert judgement. The results for the three dispersal modes (aerial dispersal of adults, aquatic up- and downstream dispersal of larvae) were then combined to give the number of dispersal modes by which each raster grid cell can be reached. To account for the uncertainty in assessing the dispersal abilities, two different scenarios were calculated for each species with cost thresholds reflecting the maximum movement distance of single individuals (progressive scenario) and the home range (conservative scenario) reported in the literature.

**Fig 3:**
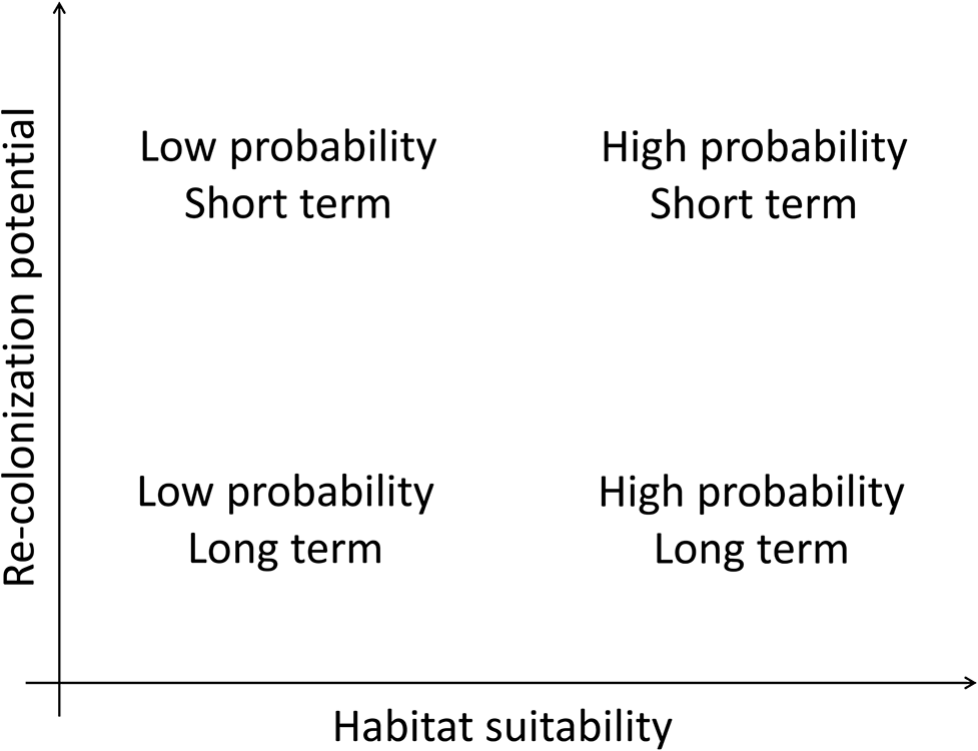
Combining results of habitat suitability and dispersal models to assess the presence of species at the reach scale.

For the **final biological assessment**, the modelling results on habitat suitability and the re-colonization potential were combined in a semi-quantitative way. Species were ranked according to the habitat suitability and re-colonization potential. Based on this ranking, they can be classified as having a high or low probability of populating the study reach (high or low habitat suitability) in the short or long term (high or low re-colonization potential) (Fig 3). The re-colonization potential was assessed for the whole river network by calculating the length of the river network that is reachable after a specific modelling period, i.e., results were upscaled to assess the effect of restoring reaches in the river network to similar habitat conditions found in the near-natural study reach. The re-colonization potential was standardized by dividing the length of the reachable river network by the total river network length, resulting in a dimensionless value (share or percentage). Similarly, values for habitat suitability were standardized. For fish, the Weighted Usable Area (WUA) was standardized by calculating its share on the bankfull wetted area, which can be considered the maximum possible value (100%). In addition to using the median WUA value of all 12 months, the range of the 12 values was calculated, with the minimum monthly value potentially acting as a bottleneck. For macroinvertebrates, the abundance predicted by the Habitat Evaluation Tool (HET) for the present substrate and nutrient conditions was standardized using the abundance predicted for the natural substrate conditions described in Pottgiesser and Sommerhäuser [46], which can be considered the maximum or best possible value (100%). Because the coverage of different substrates varies in natural rivers, ranges were used (e.g., 5–20% gravel), resulting in a range of natural reference conditions and, hence, a range of relative abundance values for the present state.

## Results

**Fig 4:**
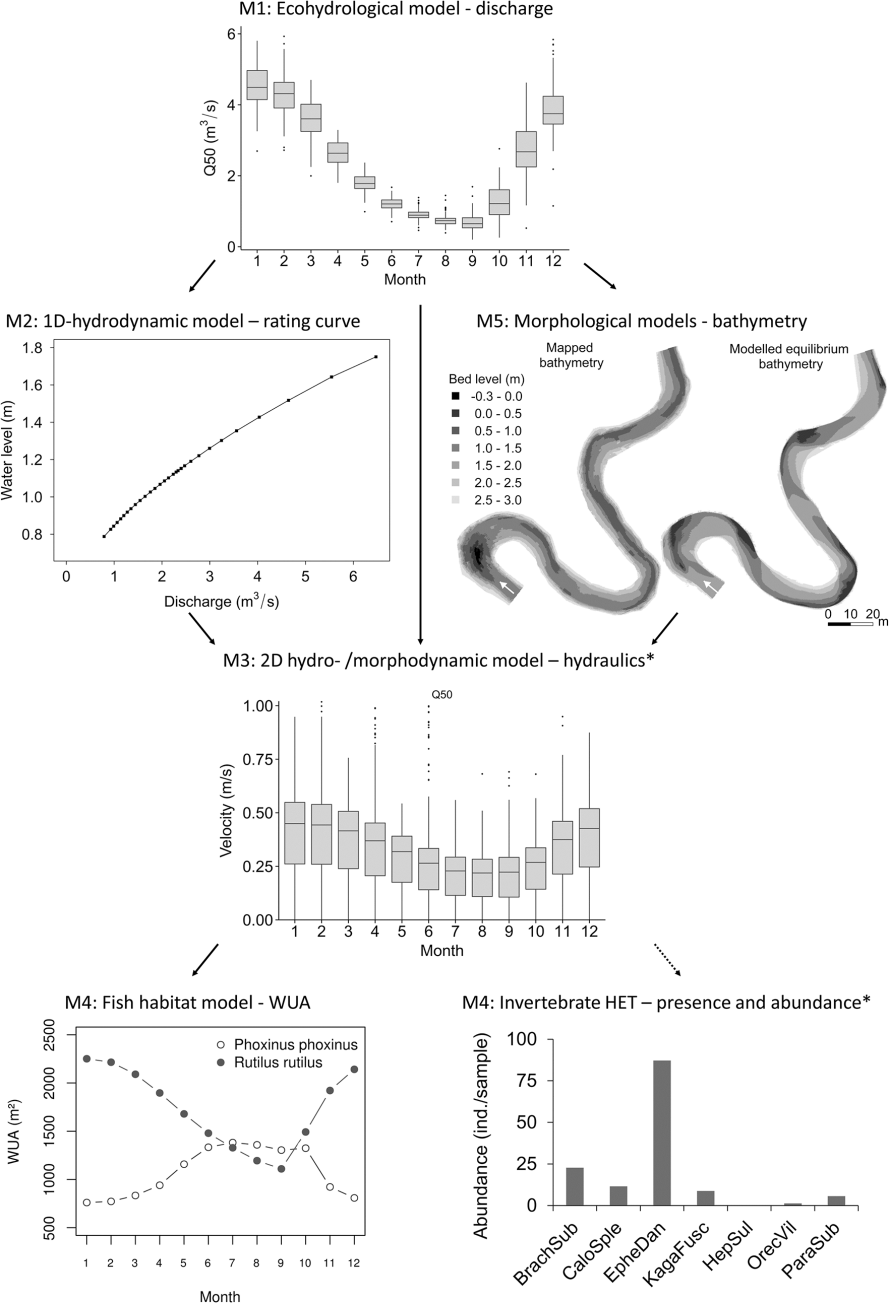
Exemplary results from the application of the modelling framework in the Treene River. The consecutive modelling steps from the ecohydrological model to the habitat models are shown. *Mapped substrate composition was used to illustrate the application of the Habitat Evaluation Tool (HET). Sediment transport was not modelled because mobile bed-substrate was purely sand, and hence, any sorting would result in no change in the HET output.

The performance of the **ecohydrological SWAT model** was good for discharge (a Nash-Sutcliffe efficiency index of 0.65 to 0.82 in the calibration and 0.58 to 0.80 in the validation period), satisfying for nitrate (0.62 in the calibration, 0.74 in the validation) and total phosphorus (0.56 and 0.37), but low for fine sediment loads (0.46 and 0.10). A more detailed temporal analysis of parameter sensitivity and model performance showed that the model results were especially sensitive to groundwater parameters, indicating that the groundwater module has the highest potential for improvement. Furthermore, model performance was highest for peak discharges and the recession phase and lower for long dry periods and the resaturation phase [75], i.e., at low discharges, which are of special importance for biota. The mean monthly values of the 100 STAR runs were used in the subsequent modelling steps (monthly Q50 values exemplarily shown in Fig 4M1).

A regime equation was used to predict **channel geometry (width)**

$$W=6.69\;Q_{10}^{0.28}$$

with W = bankfull channel width (m) and Q_10_ = value of the flow duration curve exceeding 10% of the days (m^3^/s). It showed a good fit (r^2^ = 0.91), which was comparable to other empirical equations for lowland sand-bed rivers [40,76]. However, the exponent of the equation was low (0.28) compared to other regime equations for sand- and gravel-bed rivers (usually approximately 0.5 [40,76]), resulting in a low effect of bankfull discharge on channel width. This was possibly due to the cohesive bank material and dense grassy vegetation in the river reaches mapped for the regime equation, resulting in high bank stability, and stressed the limited transferability of such empirical relationships. The width predicted by the regime equation (11.2 m, 9.6–13.5 m, 90% confidence interval) for the present Q_10_ (6.4 m^3^/s) was similar to the mapped bankfull width of 10.9 m, indicating that the channel geometry was near its dynamic equilibrium state.

The meander migration model for predicting **channel planform** accurately reproduced the locations of planform changes and predicted similar mean migration rates as observed for the calibration period (1953–2008, 5.8 cm/y compared to the observed 6.6 cm/y). Moreover, the empirical model of Julian and Torres [77] gave similar migration rates of 5.1 cm/y for comparable conditions with a high silt/clay content for the river banks (~90%) and grassy vegetation. Starting from a straight channel centreline, any disturbance of the flow that induces the formation of bars and meanders rapidly diminished downstream because the study reach is a highly damped system due to the very stable banks and small width/depth ratio. The fact that the study reach was nevertheless heavily meandering indicated that meander formation started under different conditions.

Modelled equilibrium **channel bathymetry** markedly differed from the mapped bathymetry (Fig 4M5). The mean modelled bed level was 14.1 cm higher, a deep pool mapped in the upstream part of the study reach was largely filled in the model, and modelled cross-sections were more asymmetric and had greater maximum depth. The differences indicated that the mapped bathymetry might have been in disequilibrium and not representative of the long-term channel bathymetry and habitat conditions. However, during additional field campaigns after mapping bathymetry in March 2011, visual inspection showed no major changes in channel bathymetry. It appeared most likely that the differences were due to processes that were not covered by the 2D morphodynamic model such as, for example, the effect of macrophytes on channel bathymetry and the feedback on macrophyte growth. Presently, there are first approaches that include aquatic vegetation in morphodynamic models (e.g., Van Oorschot et al. [78]), but there is still a lack of knowledge regarding the ecology and hydraulics of aquatic vegetation. Because such extensive research was beyond the scope of this study, mapped bathymetry was used for the subsequent modelling steps.

In the **2D-hydrodynamic model**, the best fit between mapped and modelled water levels (mean difference of 6 mm, max. of 13 mm) was obtained using the initial roughness value back-calculated from measured flow velocities (Manning n = 0.058), which can be considered a good fit given the mapping accuracy of 5 mm. Moreover, the maximum error of discharge (1.7%) was well below the acceptable error of 3% given in the FaSTMECH manual, and the observed flow patterns were reproduced. Hydraulic variables such as flow velocity and depth were calculated for mean monthly discharge values of Q75, Q50, and Q25, which were predicted by the ecohydrological model (values of the wetted model grid cells are exemplarily shown for flow velocity at Q50 in Fig 4M3).

The **habitat models** for fish and macroinvertebrates gave reasonable results. The WUA for large-bodied fish such as roach decreased with decreasing discharge because they prefer deep pool areas. In contrast, the WUA of small-bodied fish such as minnow increased in summer because they prefer shallow, slow flowing areas (Fig 4M4). Modelled abundances were especially high for macroinvertebrate species preferring the dominant sandy substrate (e.g., *Ephemera danica*, EpheDan in Fig 4M4). The fit and sensitivity of the HET model was investigated in a separate study in the Kielstau, a tributary of the Treene river. Model simulations reached a Renkonen Index (RI) of 56 between simulated and observed species abundance, which indicated a high agreement [66]. In contrast, there are principle limitations in assessing the performance of the fish habitat model because WUA cannot be directly translated to species presence nor compared to any equivalent field measurement.

The SDMs developed for the **dispersal modelling** generally performed well. SDMs for fish showed good to moderate cross-validated model results (mean AUC, i.e., area under receiver-operator curve, 0.79 ± SD 0.09). SDMs for macroinvertebrates even performed better, with AUC values ranging from 0.88 to 0.99. Actually validating the results of the dispersal models would need extensive field experiments, e.g., telemetric studies for fish, which was beyond the scope of this first application of the whole modelling framework.

**Fig 5:**
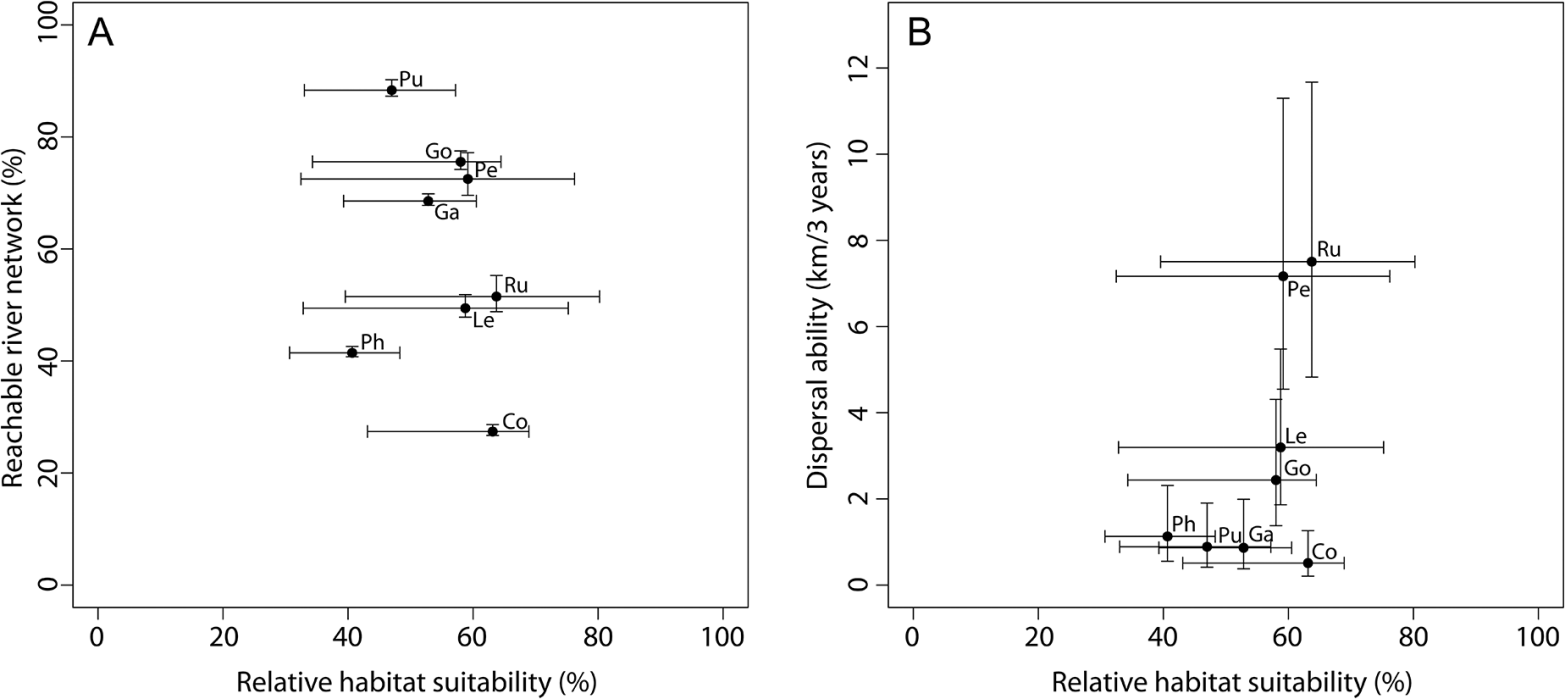
Comparison of the relative habitat suitability for fish and (A) movement distance and (B) re-colonization potential. Relative habitat suitability = WUA related to bankfull wetted area (median and range of 12 monthly values are given). Movement distance = mean and 95% confidence interval of the regression model for the mobile component in Radinger and Wolter [68]. Re-colonization potential = percentage of the river network that is reachable (95% confidence interval is given in addition). Co *Cobitis taenia*, Ga *Gasterosteus aculeatus*, Go *Gobio*, Le *Leuciscus leuciscus*, Pe *Perca fluviatilis*, Ph *Phoxinus phoxinus*, Pu *Pungitius pungitius*, Ru *Rutilus rutilus*.

For the **final assessment**, results on habitat suitability and re-colonization potential were compared. Habitat suitability of the present hydraulic habitat conditions in the study reach (baseline scenario) did not differ much between fish species (x-axis of Fig 5A and 5B). Relative habitat suitability ranged from 41% to 64% for the 8 modelled fish species (median value for all 12 months), similar to the minimum monthly value, which ranged from 31% to 43%. In contrast, fish species markedly differed in respect to their dispersal abilities and re-colonization potential. The model results reflected the different dispersal abilities, and the mean movement distance (the mean given by the regression model of the mobile component reported in Radinger and Wolter [68]) was markedly higher for good dispersers such as roach compared to less mobile species such as gudgeon (the y-axis of Fig 5A). However, fish species showed a different ranking with respect to the re-colonization potential, for which the number and location of source populations were considered in addition to species-specific dispersal abilities (y-axis of Fig 5B). Even species with a low dispersal ability such as gudgeon were predicted to reach a high share of the river network because they were widespread compared to species that are better dispersers but rare (e.g., roach).

**Fig 6:**
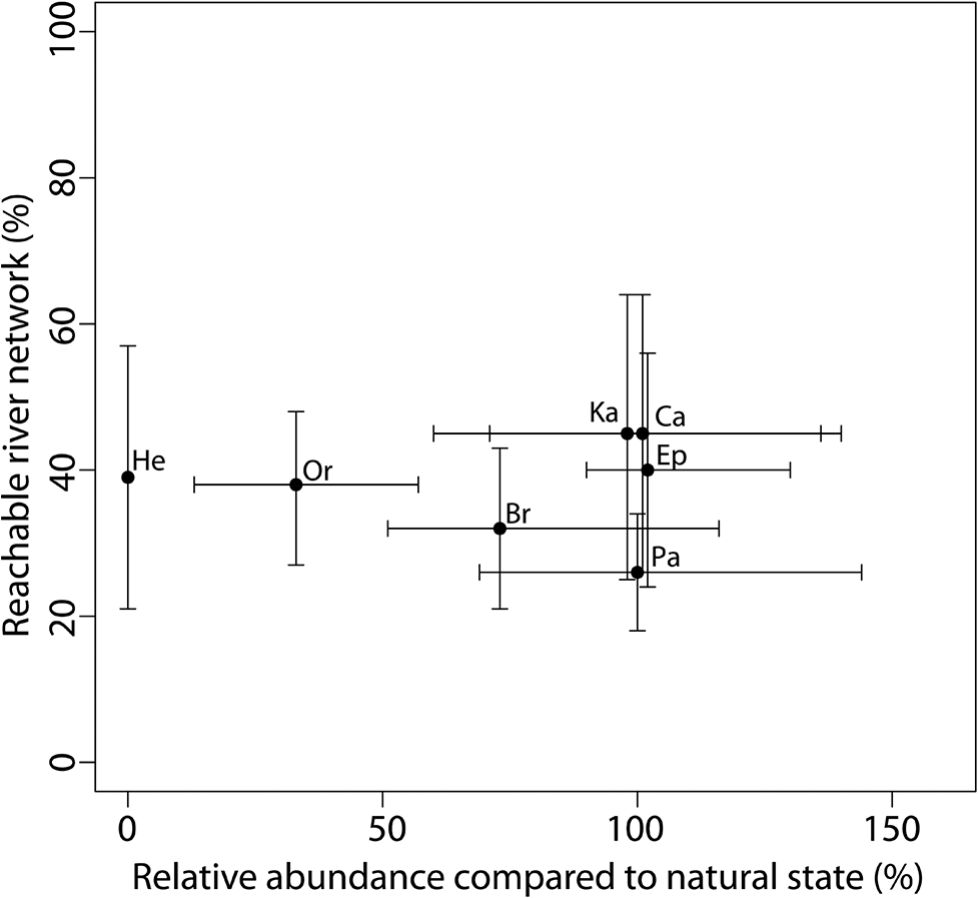
Comparison of the relative abundance of macroinvertebrates and the re-colonization potential. Relative abundance = modelled abundance for present substrate conditions related to abundance for a range of natural substrate conditions (median and range are given). Re-colonization potential = percentage of river network that is reachable (a range of conservative and progressive scenarios are given). Br *Brachycentrus subnubilus*, Ca *Calopteryx splendens*, Ep *Ephemera danica*, Ka *Kageronia fuscogrisea*, Or *Orectochilus villosus Lv*., Pa *Paraleptophlebia submarginata*, He *Hetpagenia sp*.).

For macroinvertebrates, the habitat suitability of the present substrate conditions in the study reach (baseline scenario) did substantially differ between the macroinvertebrate species (the x-axis in Fig 6). For most model species, the present substrate conditions in the study reach were equally well suited as the natural reference substrate conditions. For these species, the modelled abundance values for the present state were similar to the reference values with a median relative abundance of 98–102%. However, organic substrates and gravel would be more abundant in the natural state, and hence, the modelled abundance values of all species mainly occurring on these substrates were much lower in the baseline scenario and only reached 33–74% of the median abundances predicted for the natural reference conditions. In contrast to fish, the macroinvertebrate model species did not differ much in respect to their re-colonization potential (the y-axis in Fig 6), and the share of the river network that was reachable only ranged from 26–45%.

Based on these results (Figs 5 and 6), it was concluded that the probability of establishing in a restored reach with similar habitat conditions is similar for the modelled fish species, but they differ markedly in the time needed for colonizing the restored reach. In contrast, the modelled macroinvertebrate species mainly differ in respect to their habitat needs, and some species will be limited by the lack of adequate substrates, while they do not differ greatly in respect to the re-colonization potential, which is similarly low for all species. Moreover, the results show that the uncertainty in predicting the re-colonization potential is much higher for macroinvertebrates compared to fish (the y-range in Fig 6 compared to that in Fig 5B), which makes it difficult to detect the effect of changes, e.g., in the number of source populations or migration barriers for macroinvertebrates. However, the results indicate that the effect of substrate changes on macroinvertebrates as well as changes in habitat suitability and the re-colonization potential of fish can be investigated in different model scenarios.

## Discussion

The overall aim of this study was to further develop and test the modelling framework of Kiesel et al. [17] to assess the effect of different pressures on abiotic habitat conditions and biota of rivers. Ecohydrological, 1D hydrodynamic, morphological, and 2D hydro- and morphodynamic models as well as habitat models were linked to assess habitat suitability at the river reach scale considering environmental conditions and pressures at the catchment scale (Fig 2). This allows to predict how catchment scale changes (e.g., discharge and nutrient loads due to climate or land-use change) propagate through the model cascade down to reach-scale habitats and associated species. Furthermore, dispersal models were developed [67] and included in the modelling framework to assess and compare the importance of habitat suitability and species’ availability for re-colonization.

The case-study application for the Treene River showed that technically, it was rather simple to link the ecohydrological, 1D and 2D hydrodynamic and habitat models to assess the effects on biota and to compare them to other pressures such as water pollution or a low re-colonization potential. However, some manual steps were necessary to import and export data, which was mainly caused by some of the software tools being closed source and not based on the same scripting language. Interfaces could have been developed, but the development and use of open source software based on compatible scripting languages would greatly facilitate the automated application of such complex modelling frameworks [79], similar to the use of GRASS GIS [80] and the statistical software “R” [81] for the development of the fish dispersal model FIDIMO [67]. This would especially allow for a larger number of model runs for different scenarios and an overall uncertainty / sensitivity analysis.

From a non-technical eco-hydromorphological point of view, the following research needs were identified based on the case-study application:

In the **2D hydro- and morphodynamic model** (Fig 2M3), considering natural channel features such as boulders, large wood, and macrophytes would enable a better prediction of habitat suitability because they affect river morphology and habitat conditions (e.g., the flume experiments of Davidson and Eaton [82]). However, while boulders–which are naturally absent in the case study river–can be included [83], it is difficult to model the effect of large wood and macrophytes in 2D hydro- and morphodynamic models. Including complex shapes such as large wood would need high-resolution 3D modelling grids, resulting in an excess of computational complexity at the reach scale. Aquatic vegetation (macrophytes) probably affected channel-bathymetry in the study reach, which is consistent with effects on channel flow, morphology and biota reported in other studies [84–86]. There are first approaches that include aquatic vegetation in morphodynamic models (e.g., Van Oorschot et al. [87]), and some information on the habitat preferences of macrophytes at the reach scale are available [87–90]. However, the ability to predict macrophyte abundance and growth at the patch scale, which would have to be considered in 2D morphodynamic models, is still limited [91]. It was possible to use the present channel bathymetry mapped in the field for the baseline scenario in this study because it was near its natural dynamic equilibrium state, but changes in the channel-forming discharge in the scenario runs potentially affect bathymetry and habitat conditions. This stressed the need to develop tools to predict the presence, abundance, and growth, as well as the hydraulic and morphological effects, of macrophytes. The feedback of organism groups such as macrophytes on abiotic habitat conditions is already included in the modelling framework and represented by the arrow pointing from the biological assessment to the 2D flow and substrate conditions (Fig 2) but has not been applied in the Treene case study.

A fuzzy logic approach was used to assess **habitat suitability** for fish (Fig 2M4) due to the drawbacks of habitat preference curves outlined in the materials and methods section. However, the fuzzy logic approach was mainly based on expert judgement, which potentially introduces subjective bias due to the expertise on certain river types and degradation states, especially for widely distributed species utilizing a broad variety of waters and habitats. Moreover, the fuzzy logic habitat suitability models are usually mainly based on hydraulic and hydromorphological habitat conditions, while interacting physico-chemical variables (temperature and oxygen content) are often neglected (however, see attempts in HABITAT, http://habitat.deltares.nl). Therefore, there is a need to improve and derive empirically based rules for the fuzzy logic habitat suitability models and to include additional habitat parameters for which data are provided by the abiotic models.

The novel Habitat Evaluation Tool for macroinvertebrates is able to predict the presence and abundance of species. The modelling is based on habitat-specific sampling data to derive empirical relationships between the presence of specific habitats and species abundance. Because mainly substrate-specific biological sampling data were available, application of the Habitat Evaluation Tool has so far focused on substrate habitats, but in principle, other habitat parameters such as hydraulic variables (e.g., flow velocity, flow depth, and shear stress) could also be used. However, comparable and ecologically meaningful data on the hydraulic habitat conditions for a specific discharge (e.g., low, medium, and high flow) are rarely available for biological sampling sites because flow measurements are restricted to the arbitrary discharge that occurred during field sampling. In a recent project, hydrodynamic models are used to predict the hydraulic habitat conditions for specific discharges at a larger number of biological sampling sites to develop such habitat-specific empirical relationships, which could be used in future applications of the Habitat Evaluation Tool (http://glance-projects.eu). Furthermore, as in virtually all habitat models, (i) interactions between species such as competition, invasion, and predation were not considered [18]. Moreover, habitat models are based on empirical relationships between abiotic habitat conditions and species presence and abundance, with many abiotic variables being co-correlated (e.g., flow and substrate conditions). Thus, although the modelling framework allows to disentangle the effect of different pressures on the abiotic habitat conditions using physical-based models (e.g., climate vs. land-use changes in discharge and flow conditions), the effects of different abiotic variables on biota cannot be fully de-coupled. However, abiotic variables with rather independent state-response relationships can be investigated separately (e.g., nutrient loads and discharge).

For **morphological modelling** (Fig 2M5), three consecutive steps were used because a fully physical-based morphodynamic model to predict the dynamic equilibrium state and long-term evolution of river morphology was lacking. The channel-geometry and meander migration models were well suited to describe the overall channel morphology (e.g., mean width, depth, sinuosity, and migration rates) but did not allow modelling the natural variability in cross-section form due to the use of a mean or constant river width. Progress towards a physical model with variable width has been recently made [21]. Such models could be potentially used to model morphological changes in more detail, although practical applicability has to be tested.

In addition to the limitations of the fish **dispersal model** discussed in Radinger et al. [67], the modelling results are potentially affected by the number and location of source populations [67]. Identifying source populations based on species distribution models, such as in our case-study application, likely overestimates their number. This is in particular at sites where the selected habitat variables suggest high suitability, but neglected pressures are constraining species presence (e.g., dispersal limited by migration barriers). In contrast, empirical survey data likely underestimate the number of source populations because they rarely cover whole river networks and are usually limited to a restricted number of sampling sites. The uncertainty in quantifying the dispersal abilities of fish was low compared to the species-specific differences in the re-colonization potential (see error bars in Fig 5B). In contrast, the limited information available on the dispersal abilities of macroinvertebrates resulted in a high uncertainty of the modelling results, which was greater than the species-specific differences and made interpretation difficult. These results stressed the need to derive information on the dispersal abilities of macroinvertebrates from empirical data to allow for a more robust modelling of the re-colonization potential.

In addition to these specific limitations and research needs for the single models, an overall sensitivity analysis is an essential next step in testing the modelling framework. Moreover, transferability of the empirical morphological and biological relationships used in the case-study application is limited to similar catchments. However, data availability is rapidly increasing, for example, due to the monitoring programme of the EU Water Framework Directive (WFD). Moreover, the necessary hydrological models are set up for an increasing number of catchments for different purposes (e.g., flood protection and WFD implementation [92]), and computational power is still increasing. Therefore, our modelling framework can potentially be widely applied in the near future. Even its application in some single catchments could give important insights into many fields of river ecology and management, some of which are mentioned below.

The overall modelling framework is flexible and enables investigating different research and management options in addition to its application in climate change research: Additional pressures can easily be included given that they can be predicted by the abiotic models (e.g., water temperature changes and pollutants other than nutrients), and information on the effect on biota is available. Because hydrological, morphological, and biological processes at different spatial scales are considered, far-reaching conclusions can be drawn in river rehabilitation and management, for example, by comparing the (cost) effectiveness of measures implemented at different spatial scales (e.g., reach-scale instream habitat measures, development of riparian buffer strips at the river network scale, and catchment scale land-use changes).
